# Feasibility of a rapid C-reactive protein chairside point-of-care test for detecting inflammation in exposed dental pulp: a pilot exploratory study

**DOI:** 10.1038/s41405-025-00340-w

**Published:** 2025-05-23

**Authors:** Mohamed Taha Elfezary, Ahmed Safaa Waly, Eman Hamdy Mohamed

**Affiliations:** 1 Department of Pediatric Dentistry, Faculty of Dentistry, Al-Azhar University, Assiut, Egypt; 2 Department of Clinical and Chemical Pathology, Faculty of Medicine, Beni-Suef University, Beni-Suef, Egypt

**Keywords:** Paediatric dentistry, Pulpitis, Pulp conservation

## Abstract

**Objective/Aim:**

Dental pulp inflammation is a critical condition in endodontics. Traditional diagnostic methods, such as patient pain history and percussion tests, often lack accuracy in reflecting the true status of pulp inflammation. This study explores the feasibility of using a rapid C-reactive protein (CRP) chairside point-of-care (POC) test as a potential adjunctive tool for detecting dental pulp inflammation. The findings provide preliminary insights to inform future larger-scale validation studies.

**Materials and methods:**

This pilot cross-sectional observational study included 20 patients with deep carious lesions. Blood samples were collected from exposed pulp tissue under sterile conditions and analyzed using the CRP rapid POC test (index test). Patient pain history (clinical reference test) and percussion test outcomes were documented. The study assessed feasibility and preliminary diagnostic trends based on test performance and associations with clinical indicators.

**Results:**

The CRP rapid POC test yielded positive results in 55% of cases. Preliminary findings suggest a potential association between CRP levels and percussion test results (*p *< 0.001), while no significant correlation was observed between CRP levels and patient pain history. The test demonstrated an observed sensitivity of 94.3%, specificity of 87.1%, positive predictive value (PPV) of 90.7%, and negative predictive value (NPV) of 91.9%. However, given the small sample size, these estimates should be interpreted with caution, and further research with larger cohorts is necessary for validation.

**Conclusion:**

This pilot study suggests that the CRP rapid POC test may have potential as a diagnostic aid for detecting dental pulp inflammation. However, these findings are preliminary, and further validation through larger studies and gold-standard comparisons is necessary before clinical implementation can be considered.

## Introduction

Dental pulp inflammation presents a critical diagnostic challenge in endodontics [1]. Accurate diagnosis is essential for determining whether inflammation is reversible, allowing conservative treatment, or irreversible, necessitating root canal therapy [2]. Current diagnostic practices, including pain history and percussion tests, rely heavily on subjective assessments, often failing to accurately reflect the pulp’s condition [3]. These methods lack sensitivity and specificity, leaving a gap in reliable diagnostic tools to guide clinical decision-making [4].

Histopathological analysis and enzyme-linked immunosorbent assay (ELISA) are considered gold standards for diagnosing pulp inflammation [5]. However, their high cost, time consumption, and requirement for specialized personnel render them impractical for routine clinical use [6]. This limitation has driven the need for simpler, more accessible diagnostic alternatives. Rapid point-of-care (POC) tests, such as those measuring C-reactive protein (CRP) levels, have demonstrated potential in systemic inflammatory conditions and periodontal diseases [7–9]. CRP, an acute-phase protein, is a nonspecific marker of inflammation produced in response to tissue injury or infection [10]. Its application in dental pulp diagnostics remains underexplored but holds promise for chairside use due to its rapidity and simplicity [https://biopanda.co.uk/php/products/rapid/crp.php].

The scientific rationale for evaluating CRP in this context stems from its ability to detect localized dental pulp inflammation [10]. By correlating CRP levels with clinical indicators such as pain history and percussion test results, we hypothesize that CRP testing can offer an objective measure of pulpitis severity. Specifically, this study seeks to fill the knowledge gap regarding the feasibility and diagnostic accuracy of CRP rapid POC testing in detecting dental pulp inflammation.

### Aim

The aim of this pilot study was to assess the feasibility of using a CRP rapid POC test as a potential diagnostic aid in distinguishing between reversible and irreversible pulpitis. This study provides preliminary insights that may inform future, larger-scale validation studies.

#### Hypothesis

We hypothesize that the CRP rapid POC test (Index test) may demonstrate potential diagnostic value in detecting dental pulp inflammation. Given the exploratory nature of this study, findings will be interpreted cautiously, with the goal of informing future research rather than establishing definitive diagnostic accuracy.

## Materials and methods

### Study design

This diagnostic cross-sectional observational pilot study evaluated the feasibility of using the CRP POC test for detecting dental pulp inflammation. Given the exploratory nature of the research, this study does not aim to establish definitive diagnostic accuracy but rather to provide preliminary insights to inform future larger-scale investigation.

### Ethical consideration

The study received ethical approval from the Faculty of Dental Medicine’s Institutional Review Board (AUAREC20230006-5), in line with the Preferred Reporting Items for Diagnostic Accuracy Studies in Endodontics (PRIDASE) guidelines for reporting diagnostic accuracy studies specific to endodontic diagnosis. The PRIDASE guidelines were developed based on STARD 2015 principles, ensuring compliance with the broader STARD framework. Informed consent was obtained from all participants.

### Inclusion criteria

- Patients aged 5 years or older with deep caries in primary or permanent teeth.
- Pain history (Chief complaint) suggesting reversible or irreversible pulpitis (Target condition) based on criteria of American Association of Endodontic (AAE) [endodonticdiagnosisfall2013.pdf].
- No prior use of nonsteroidal anti-inflammatory drugs.
- Willingness to provide informed consent.

### Exclusion criteria

- Teeth with no observable bleeding during the procedure (indicative of necrotic or nonvital pulp and calcified pulp).
- Patients with systemic conditions are known to affect CRP levels.
- Teeth exhibiting periodontal disease as aggressive periodontitis [9] or radiographic signs of periapical pathosis.

### Patient recruitment

Participants were recruited consecutively from outpatient’s dental clinic and private clinic between [July/2023] and [May/2024] based on their presenting symptoms. Eligibility was determined using a combination of medical and dental history, chief complaint, clinical examination, and periapical testing. based on criteria of (AAE), key diagnostic steps included evaluating [endodonticdiagnosisfall2013.pdf]:

- Medical/dental history: past and present
- Chief complaint: pain onset, duration, and stimuli; location, relief factors, for pediatric participants, pain history was obtained through parental reporting.
- Clinical examination: Facial symmetry, sinus tract presence, periodontal probing, caries, and restoration status.
- Periapical tests: Percussion, palpation.
- Radiographic analysis. Deep caries close or into pulp were included in the study.

The classification of pulpal inflammation (reversible vs. irreversible pulpitis) was determined based on AAE guidelines [endodonticdiagnosisfall2013.pdf], incorporating medical and dental history, clinical examination, periapical testing, and radiographic analysis. However, for the purpose of this study, patient pain history was used as the primary clinical reference test to classify pulpitis. The CRP test was not used to diagnose or classify pulpitis; it was evaluated solely as a potential adjunctive tool for feasibility assessment.

### Study procedures (CRP measurement)

A single investigator performed all procedures to minimize variability, achieving an intraexaminer agreement (Cohen’s kappa = 0.9). Local anesthesia (4% articaine with 1:100,000 epinephrine) was administered before caries removal.

- **Deep caries without pulp exposure:** For cases where pulp exposure did not occur, indirect pulp capping was performed, and these patients were excluded.
- **Deep caries with pulp exposure:** In cases meeting criteria for pulp exposure, the roof of the pulp chamber was removed, and patients without observable blood were excluded.

**Fig. 1:**
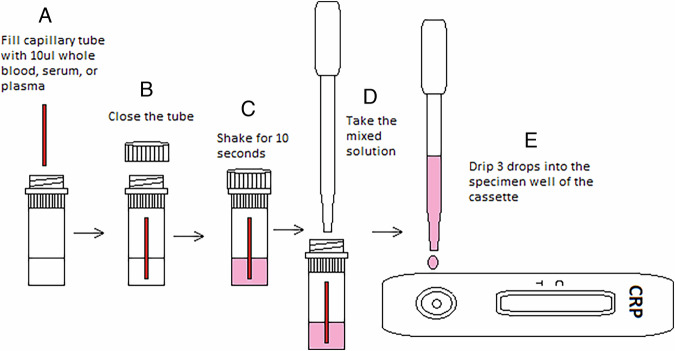
Workflow for preparing and applying the blood sample to the semiquantitative CRP rapid‑test cassette (image reproduced with permission from Biopanda Reagents, England). **A** A 10 μL capillary tube is filled with whole blood, serum, or plasma. **B** The tube is closed with its cap. **C** The tube is inverted and shaken vigorously for 10 seconds to mix the sample. **D** The mixed pink solution is drawn back into the capillary tube. **E** Three drops of the sample are dispensed into the specimen well of the CRP test cassette. Notes: The red line in the capillary tube indicates the 10 μL volume mark; pink coloration represents the mixed sample ready for testing. [https://biopanda.co.uk/php/products/rapid/crp.php].

**Fig. 2:**
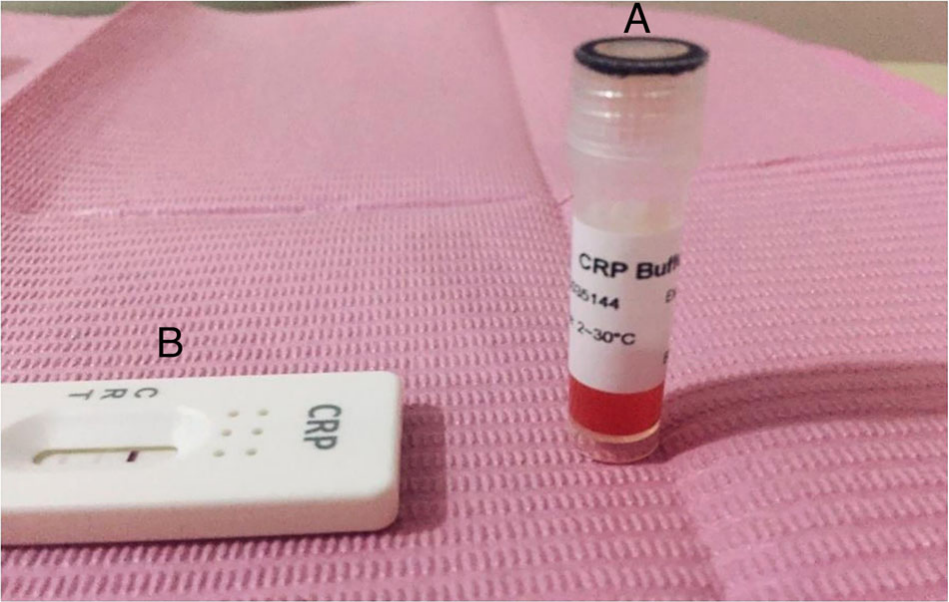
CRP rapid‑test buffer solution and test cassette. A single panel shows **A** the buffer vial (red solution) labeled “CRP Buffer” used to reconstitute or dilute the sample, placed next to **B** the plastic CRP test cassette with its specimen well and result window. Notes: The red‑colored buffer provides the necessary reaction environment for the assay; the cassette is marked with “C” (control line) and “T” (test line) positions for result interpretation.

Under rubber dam isolation approximately 10 µL of blood was collected using a capillary tube or sterile cotton piece [11], mixed with a CRP buffer solution as per the manufacturer’s instructions (CRP Rapid Test, Biopanda Reagents, UK). The mixture was shaken vigorously for 10 s before three drops (120 µL) were placed on the test cassette (Figs. 1 and 2). Results were interpreted within 5 min, disregarding any changes observed after 10 min. CRP point-of-care test with manufacturer-defined cut-off values, where levels below 10 mg/ml were considered negative. These cut-off points are well-validated for systemic inflammation and were applied in our study to evaluate the feasibility of using CRP as a marker for dental pulp inflammation [https://biopanda.co.uk/php/products/rapid/crp.php].

### Reference standard and sample size

Due to ethical and practical reasons, no normal pulp samples or gold-standard test (e.g., ELISA or histopathology) were used [12]. Instead, the patient’s pain history (chief complaint) served as clinical reference standard which is the best available method for establishing the presence or absence of the target condition. These criteria have been validated in prior studies, which confirmed a high degree of concordance between such diagnostic methods and histologic/histobacteriologic analyses [13, 14]. This validation reinforces the reliability of our approach, despite the lack of histology as a gold standard.

This study is a pilot exploratory investigation with a sample size of 20 patients. As this is a feasibility study, no formal sample size calculation was performed, and the sample size was selected based on practical considerations to provide preliminary insights for future larger-scale studies [15].

### Statistical analysis

Descriptive statistics (frequencies, percentages, means, and standard deviations) were calculated for demographic and clinical variables. As this is a pilot feasibility study, diagnostic performance metrics, including sensitivity, specificity, positive predictive value (PPV), and negative predictive value (NPV), were explored using Latent Class Analysis (LCA), which is appropriate for studies with an imperfect reference standard. Given the small sample size, these estimates should be interpreted as preliminary and hypothesis-generating rather than definitive accuracy measures.

CRP levels were categorized as binary (negative/positive) for exploratory diagnostic analysis and ordinal (Reversible pulpitis: CRP < 10 mg/ml, mild to moderate irreversible pulpitis: CRP 10–30 mg/ml, severe irreversible pulpitis: CRP > 30 mg/ml) for further statistical evaluation. Mann–Whitney U test and ordinal logistic regression analyses were performed to assess associations between CRP levels and clinical indicators, with statistical significance set at *p *< 0.05. All analyses were conducted using SPSS version 29.

## Results

### Demographic characteristics

The study sample comprised 9 males (45%) and 11 females (55%), with ages ranging from 5 to 40 years (mean age: 21.9 ± 11.3 years). Based on age grouping, six participants (30%) were under 13 years of age (pediatric), and 14 participants (70%) were adolescents or adults. The majority of participants (*n* = 18, 90%) had permanent teeth, while two cases (10%) involved primary teeth (Table 1).

**Table 1:** Demographic characteristics.

Variable	Category	Count (*n* = 20)	Percentage (%)
Gender	Male	9	45%
	Female	11	55%
Age (years)	5–12 (Pediatric)	6	30%
	≥13 (Adolescent & Adult)	14	70%
	Min–Max	5–40	–
	Mean ± SD	21.9 ± 11.3	–
Tooth type	Primary	2	10%
	Permanent	18	90%

### Preliminary diagnostic findings

The study included 20 participants, all of whom underwent the CRP rapid POC test (index test). Among them, 9 tested negative and 11 tested positive.

**Fig. 3:**
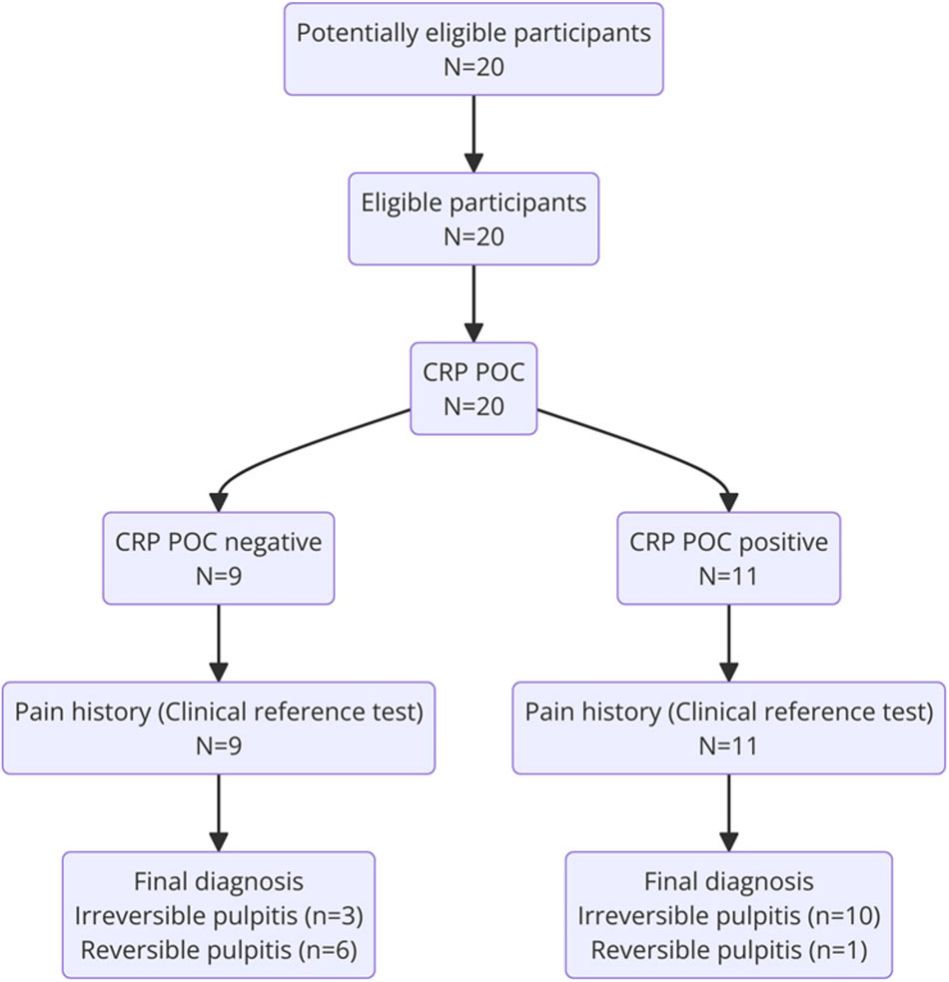
Participant flow diagram per STARD 2015. Twenty potential participants were screened and all (*N* = 20) underwent CRP point‑of‑care (POC) testing: nine were CRP‑negative (→ pain‑history reference → 3 irreversible pulpitis, 6 reversible pulpitis) and eleven were CRP‑positive (→ pain‑history reference → 10 irreversible pulpitis, 1 reversible pulpitis).

For participants with a negative index test, the clinical reference standard confirmed the presence of irreversible pulpitis in 3 cases and its absence in 6 cases. For participants with a positive index test, the clinical reference standard confirmed irreversible pulpitis in 10 cases and its absence in 1 case (Fig. 3).

The distribution of pain history and CRP POC test results were analyzed. Among patients diagnosed with reversible pulpitis, 6 (85.7%) had a negative CRP POC test result, while 1 (14.3%) tested positive. In cases of irreversible pulpitis, 3 (23.1%) had a negative test result, while 10 (76.9%) tested positive. Overall, the CRP POC test identified 9 patients (45.0%) as negative and 11 patients (55.0%) as positive across the study population (Table 2).

**Table 2:** Cross tabulation of pain history and CRP POC test results in diagnosing pulpitis.

			CRP POC^b^		
			Negative	Positive	Total
Pain history^a^	Reversible	Count %	6 (85.7%)	1 (14.3%)	7 (100%)
		% within CRP POC	66.7%	25%	35%
	Irreversible	Count %	3 (23.1%)	10 (76.9%)	13 (100%)
		% within CRP POC	33.3%	75%	65%
Total		Count %	9 (45%)	11 (55%)	20 (100%)
		% within CRP POC	100%	100%	100%

^a^Clinical reference standard: the best available method for establishing the presence or absence of the target condition.

^b^Index test.

### Exploratory diagnostic performance analysis

**Fig. 4:**
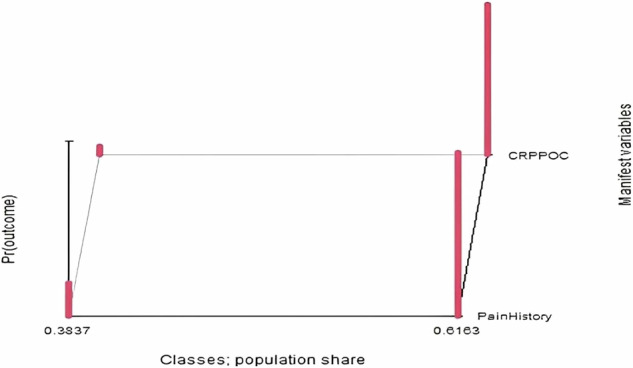
Probability distribution of diagnostic outcomes for pain history and CRP point-of-care (CRP POC). The x-axis (“Classes: population share”) shows the two classes: pain history (population share 0.6183) and CRPPOC (population share 0.3837). The y-axis (“Pr(outcome)”) represents the probability of a positive outcome. Classification metrics for pain history versus CRP POC. Bars show the proportion of true positives (TP), true negatives (TN), false positives (FP), and false negatives (FN) for each test: blue = pain history; orange = CRP POC.

The CRP rapid POC test was compared with pain history as a reference standard using Latent Class Analysis (LCA) (Table 3, Fig. 4).

**Table 3:** Class proportions and conditional probabilities.

Class	Proportion (percentage)	Pain history (1)	Pain history (2)	CRP POC (1)	CRP POC (2)
Class 1 (Negative)	0.384 (38.4%)	0.809	0.064	0.948	0.140
Class 2 (Positive)	0.616 (61.6%)	0.191	0.936	0.052	0.860

**Fig. 5:**
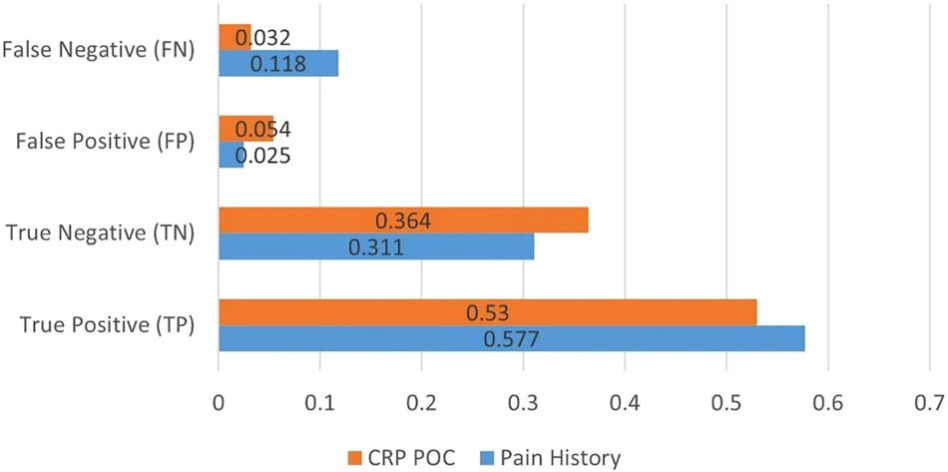
Classification metrics for Pain History versus CRP POC. Bars show the proportion of True Positives (TP), True Negatives (TN), False Positives (FP), and False Negatives (FN) for each test: blue = Pain History; orange = CRP POC.

The true positive rate for pain history was 0.577, while the CRP POC test had a true positive rate of 0.530. Notably, the CRP POC test demonstrated a lower false-negative rate (0.032) than pain history (0.118), suggesting that the test may have potential in reducing overlooked cases of pulp inflammation (Fig. 5).

Preliminary findings indicate that the CRP POC test yielded an observed sensitivity of 94.3% and specificity of 87.1%, with a positive predictive value (PPV) of 90.7% and a negative predictive value (NPV) of 91.9%. However, given the small sample size, these estimates should be interpreted cautiously, and further studies are needed to validate diagnostic accuracy in larger populations (Table 4).

**Table 4:** Sensitivity, specificity, and predictive value of pain history and CRP POC detection.

Metric	Value (Pain history)	95% CI (pain history)	Value (CRP POC)	95% CI (CRP POC)
Sensitivity	83.0%	(14.7–99.3)	94.3%	(18.1–99.9)
Specificity	92.6%	(18.2–99.9)	87.1%	(15.5–99.6)
PPV	95.8%	(19.4–100)	90.7%	(16.8–99.8)
NPV	72.5%	(11.3–98.2)	91.9%	(17.2–99.8)
Accuracy	88.8%	(16.7–99.7)	91.5%	(17.0–99.8)

### Associations of pain history and percussion test results with CRP POC levels

Significant associations emerged between pain history and CRP POC levels. Patients diagnosed with reversible pulpitis demonstrated a high prevalence of low CRP expression, with 85.7% showing negative CRP results. In contrast, among irreversible pulpitis cases, only 23.1% had negative CRP results, while 53.8% exhibited mild to moderate CRP levels, and 23.1% had severe CRP elevation (*p *= 0.033).

Percussion test results revealed a stronger association with CRP POC levels. Among patients with a negative percussion test, 81.8% had negative CRP results, while 66.7% of those with a positive percussion test exhibited mild to moderate CRP levels, and 33.3% had severe CRP elevation (*p *< 0.001).

**Fig. 6:**
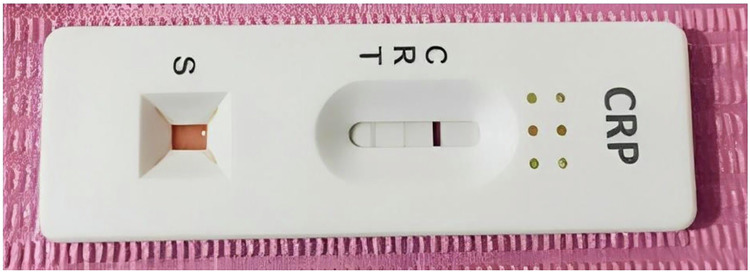
Positive CRP POC test result (30 mg/L) in a patient with irreversible pulpitis. The lateral‐flow cassette displays a clear control line (C) and a distinct test line (T), indicating a CRP concentration above 30 mg/L.

Regression analysis showed that pain history (coefficient = 0.499, *p *= 0.753) was not significantly correlated with CRP levels. However, a positive percussion test was significantly correlated with higher CRP levels (coefficient = 3.484, *p *= 0.025), suggesting that patients with a positive percussion response may have greater inflammatory severity (Table 5, Fig. 6).

**Table 5:** Regression analysis of clinical predictors for CRP POC levels in pulpitis patients.

Predictor	Coefficient	*p* value	95% Confidence interval
Pain History (Irreversible)	0.499	0.753	−2.60–3.60
Percussion Test (Positive)	3.484	0.025	0.44–6.52

## Discussion

This pilot study explored the feasibility of using a CRP rapid POC test as a potential adjunctive tool for detecting dental pulp inflammation. The findings suggest that the test may offer preliminary insights into inflammation status, which could help refine diagnostic strategies in endodontics. However, given the small sample size, these results should be interpreted cautiously, and further validation in larger, controlled studies are needed.

Although evidence suggests that vital pulp therapy may be effective even in cases of irreversible pulpitis [16], the supporting data remains weak or of low quality. Consequently, many dentists favor root canal treatment due to its more predictable outcomes. Current diagnostic methods for assessing pulp inflammation severity and distinguishing between reversible and irreversible pulpitis rely largely on subjective evaluation, which lacks reliability. Given the critical role of accurate diagnosis in the success of vital pulp therapy [17], the CRP POC test offers a more objective and precise approach.

The CRP test allowed for a semiquantitative grading of inflammation, categorizing cases into no, mild to moderate, or severe inflammation. This preliminary classification system may support clinical decision-making and potentially assist in differentiating between reversible and irreversible pulpitis. However, due to the study’s exploratory nature, these findings should be validated against gold-standard diagnostic methods such as histopathology or ELISA.

Compared to subjective diagnostic methods like patient-reported pain history, the CRP test demonstrated preliminary diagnostic performance trends, particularly in sensitivity and NPV. This study observed that the CRP test had an estimated sensitivity of 94.3% and NPV of 91.9%, suggesting it may help reduce false-negative diagnoses. However, given the small sample size and absence of a gold standard, these estimates must be interpreted with caution and should not be used to make definitive clinical recommendations.

Pain history exhibited a slightly higher specificity (92.6%) and PPV (95.8%), indicating its potential for confirming inflammation when present. However, subjective assessments depend on individual pain perception, which introduces variability. In this study, thermal tests were not used, as they primarily assess neural response rather than vascular inflammation [4], which is a limitation to consider.

This study employed Latent Class Analysis (LCA) to assess the preliminary diagnostic performance of the CRP test, as gold-standard validation (e.g., histopathology or ELISA) was not feasible due to ethical and practical constraints [12]. While LCA provides a probabilistic approach to diagnostic evaluation [6], it cannot replace direct validation against a confirmed pathological diagnosis. Future studies should aim to validate these findings with a larger sample size and gold-standard comparisons.

Our findings align with prior research by Proctor et al., who demonstrated that CRP levels in inflamed dental pulp are localized rather than influenced by systemic CRP levels [10]. This reinforces the potential feasibility of using a chairside CRP POC test to provide real-time clinical insights [https://biopanda.co.uk/php/products/rapid/crp.php]. Similar rapid tests have been explored in periodontal disease [7, 8] and systemic inflammation [18], further supporting the potential for molecular-based diagnostic tools in dental practice.

The observed lack of significant correlation between CRP levels and patient pain history in regression analysis raises questions about pain history’s reliability as a diagnostic tool. Variability in pain perception and reporting among patients may contribute to inconsistencies in clinical diagnosis [4, 16]. However, the strong association between percussion test results and CRP levels (*p *< 0.001) suggests that percussion testing may better reflect localized inflammatory severity [19, 20].

Findings by Garrido et al. demonstrated elevated CRP levels in apical lesions of endodontic origin [21], further supporting the hypothesis that CRP is a localized inflammatory marker. However, periapical pain on percussion can also result from periodontal conditions [20], indicating that CRP testing should not be used in isolation but rather as an adjunct to other diagnostic tools.

Demographic diversity, including age and dentition type, may influence pulp response. This pilot study included only two cases (10%) with primary teeth and six patients (30%) under 13 years of age, which may introduce variability in tissue response or symptom reporting. To address this, pain history for pediatric participants was collected via structured parental reporting, a validated method in pediatric dental research [22]. Although we assessed local CRP expression, WHO data indicate that serum CRP levels are generally stable across age and sex [23]. While further research is needed, this suggests a low likelihood of systemic bias. Future studies should stratify by age and dentition to validate these findings.

### Limitations

Several limitations must be acknowledged:

1. Small sample size: As a pilot study, the limited sample size restricts the generalizability of the results and increases the potential for statistical variability. Wider confidence intervals further emphasize the need for larger-scale studies.
2. Age and dentition variability: The inclusion of pediatric participants and primary teeth introduces potential biological variability in pulp tissue characteristics and symptom expression. Although the number of primary teeth cases was limited (10%), and only 30% of participants were under 13 years of age, this heterogeneity may affect generalizability. Future studies should consider age and dentition-stratified analyses to ensure diagnostic accuracy across developmental stages.
3. Semi-quantitative test: While the CRP test provides greater utility than purely qualitative methods, it lacks the precision of fully quantitative assays. This may introduce ambiguity, particularly near the 30 mg/ml threshold, which could affect clinical interpretation.
4. Imperfect reference standard: The reliance on pain history as a clinical reference introduces subjective bias, as pain perception varies among individuals and may not reliably indicate pulpal inflammation severity.
5. Lack of negative controls: Ethical considerations prevented the inclusion of negative control samples (healthy pulp), which could have strengthened diagnostic comparisons.

### Clinical implications

This pilot study suggests that the CRP rapid POC test may have potential as an adjunctive tool for assessing dental pulp inflammation. However, these findings are preliminary, and further large-scale studies with gold-standard validation are necessary before clinical recommendations can be made. If validated, this test could complement existing diagnostic methods and provide real-time objective insights into pulp inflammation severity.

### Future directions

To strengthen the evidence base, future research should:

1. Increase sample size: Conduct larger, multi-center studies to improve the generalizability and reliability of findings.
2. Gold-standard validation: Compare CRP POC test results with histopathology or ELISA to establish true diagnostic accuracy.
3. Integration of additional biomarkers: Explore other inflammatory markers (e.g., matrix metalloproteinases, cytokines) to improve diagnostic precision.
4. Evaluate cost-effectiveness & clinical workflow: Assess the test’s practicality in real-world dental settings and its impact on treatment planning and patient outcomes.

## Conclusion

This pilot study provides preliminary evidence supporting the feasibility of the CRP rapid POC test as a potential adjunctive tool for assessing dental pulp inflammation. While the findings suggest that the test may assist in distinguishing between reversible and irreversible pulpitis, the small sample size and exploratory nature of the study necessitate further validation. Larger-scale studies incorporating gold-standard diagnostic methods are required to confirm these findings and refine the test’s clinical applicability.

## Data Availability

The corresponding author can provide the data upon request.
